# Development of a Multiplex qPCR Assay for Fast Detection and Differentiation of Paracoccidioidomycosis Agents

**DOI:** 10.3390/jof9030358

**Published:** 2023-03-15

**Authors:** Breno Gonçalves Pinheiro, Ana Paula Pôssa, Giannina Ricci, Angela Satie Nishikaku, Ferry Hagen, Rosane Christine Hahn, Zoilo Pires de Camargo, Anderson Messias Rodrigues

**Affiliations:** 1 Laboratory of Emerging Fungal Pathogens, Department of Microbiology, Immunology, and Parasitology, Discipline of Cellular Biology, Federal University of São Paulo (UNIFESP), São Paulo 04023-062, Brazil; 2 Department of Medicine, Discipline of Infectious Diseases, Federal University of São Paulo (UNIFESP), São Paulo 04023-062, Brazil; 3 Centro de Diagnóstico e Pesquisa em Biologia Molecular Dr. Ivo Ricci, São Carlos 13561-020, Brazil; 4 Department of Medical Mycology, Westerdijk Fungal Biodiversity Institute, Uppsalalaan 8, 3584 CT Utrecht, The Netherlands; 5 Institute for Biodiversity and Ecosystem Dynamics (IBED), University of Amsterdam, Sciencepark 904, 1098 XH Amsterdam, The Netherlands; 6 Department of Medical Microbiology, University Medical Center Utrecht, Heidelberglaan 100, 3584 CX Utrecht, The Netherlands; 7 Laboratory of Mycology/Research, Faculty of Medicine, Federal University of Mato Grosso, Cuiabá 78060-900, Brazil; 8 Júlio Muller University Hospital, Federal University of Mato Grosso, Cuiabá 78048-902, Brazil

**Keywords:** qPCR, endemic mycosis, systemic mycosis, FFPE, paracoccidioidomycosis, *Paracoccidioides brasiliensis*, *Paracoccidioides americana*, *Paracoccidioides restrepiensis*, *Paracoccidioides venezuelensis*, *Paracoccidioides lutzii*

## Abstract

Classic paracoccidioidomycosis (PCM) is a potentially deadly neglected tropical systemic mycosis caused by members of the *Paracoccidioides brasiliensis* complex (*P. brasiliensis s. str.*, *P. americana*, *P. restrepiensis*, and *P. venezuelensis*) and *P. lutzii*. The laboratorial diagnosis of PCM relies on observing pathognomonic structures such as the “steering wheel” or “Mickey Mouse” shape in the direct mycological examination, fresh biopsied tissue in 10% KOH, histopathological analysis, and/or the isolation of the fungus in culture. However, these procedures are time-consuming and do not allow for the speciation of *Paracoccidioides* due to overlapping morphologies. Here, we propose a new one-tube multiplex probe-based qPCR assay to detect and recognize agents of the *P. brasiliensis* complex and *P. lutzii*. Primers (Paracoco-F and Paracoco-R) and TaqMan probes (PbraCx-Fam, Plu-Ned, and Paracoco-Vic) were developed to target the rDNA (ITS2/28S) in the *Paracoccidioides* genome. A panel of 77 *Paracoccidioides* isolates revealed a 100% specificity (AUC = 1.0, 95% CI 0.964–1.000, *p* < 0.0001) without cross-reacting with other medically relevant fungi or human and murine DNA. The lower limit of detection was 10 fg of gDNA and three copies of the partial rDNA amplicon. Speciation using qPCR was in perfect agreement with AFLP and *TUB1*-RFLP markers (kappa = 1.0). As a proof of concept, we assessed a panel of 16 formalin-fixed and paraffin-embedded specimens from histopathologically confirmed PCM patients to reveal a significant sensitivity of 81.25% and specificity of 100% (AUC = 0.906 ± 0.05, 95% CI = 0.756–0.979, *p* < 0.0001, Youden index J = 0.8125). Our assay achieved maximum sensitivity (100%) and specificity (100%) using fresh clinical samples (n = 9) such as sputum, bronchoalveolar lavage, and tissue fragments from PCM patients (AUC = 1.0, 95% CI 0.872–1.000, *p* < 0.0001, Youden index J = 1.0). Overall, our qPCR assay simplifies the molecular diagnosis of PCM and can be easily implemented in any routine laboratory, decreasing a critical bottleneck for the early treatment of PCM patients across a vast area of the Americas.

## 1. Introduction

In recent years, a great deal of diversity has been described among *Paracoccidioides* species, and there is an intense debate about how such diversity translates into taxonomic changes [1,2]. In 2009, the classic *Paracoccidioides brasiliensis* was split into the *P. brasiliensis* complex and *P. lutzii* [3,4]. The *P. brasiliensis* complex defines a monophyletic clade harboring four cryptic species (*P. brasiliensis sensu stricto*, *P. americana*, *P. restrepiensis*, and *P. venezuelensis*) with equivalent clinical relevance [5,6]. The recent discovery of *P. cetii* (a sister species of the *P. brasiliensis* complex), which is associated with infections in dolphins, and the reclassification of the ancient *P. loboi* (formerly *Lacazia loboi*), which causes subcutaneous mycoses in humans, brought to the genus two culture-resistant pathogens, delineating seven species in a genus formerly assumed to be monotypic [1,7].

The interaction of *Paracoccidioides* species with the warm-blooded vertebrate host leads to the development of paracoccidioidomycosis (PCM), a mycosis ranging from systemic to subcutaneous disease [1]. The classic PCM described in 1908 by Adolpho Lutz remains a life-threatening systemic granulomatous disease acquired by the inhalation of propagules from members of the *P. brasiliensis* complex and *P. lutzii* [8]. However, *P. loboi* (PCM loboi) and *P. cetii* (PCM ceti) are the culprits of a subcutaneous mycosis caused by implantation, which is characterized by a wide variety of cutaneous lesions, particularly keloidiform nodes [7].

Molecular epidemiology shows that most *Paracoccidioides* species occur in sympatry with a predominant agent in more than 80% of cases [9]. For example, *Paracoccidioides brasiliensis s. str.* is the leading agent in Brazil, Argentina, Bolivia, Paraguay, Peru, Uruguay, Venezuela, and the Guadeloupe Islands. To a lesser extent, *P. americana* is described in Argentina, Brazil, Uruguay, and Venezuela. The two sister species *Paracoccidioides restrepiensis* and *P. venezuelensis* are autochthonous to Colombia and Venezuela, respectively, with sporadic cases reported from Argentina, Brazil, Peru, and Uruguay. *Paracoccidioides lutzii* has an elevated disease burden in the Brazilian Midwest, as well as scattered cases in the Amazon and Peru [4,9,10,11]. *Paracoccidioides cetii* from cetaceans is detected through the nearshore zones of the Americas, while *P. loboi* predominates in riverside populations in the Amazon basin and South America [1,7,12].

PCM diagnosis follows clinical suspicion and laboratory analysis. Direct mycological examination of fresh biopsied tissue, sputum, or pus from infected individuals reveals *P. brasiliensis* complex and *P. lutzii* multiple budding yeast-like cells producing “steering wheels”, typically pathognomonic in classic PCM. Cultures of clinical specimens showing thermal dimorphism are the reference diagnosis and permit a generic identification [13]. However, a major drawback is that *P. brasiliensis* complex and *P. lutzii* have a fastidious nature growing in vitro, taking up to 30 days for colonies to appear [8]. The resistance to culture observed in *P. cetii* and *P. loboi* is a symplesiomorphy that demands direct mycological examination and histopathology to detect chains of even-sized yeast-like cells (7–25 μm) in laboratory diagnosis [7].

Conventional methods can be challenging when identifying and distinguishing agents from classic PCM due to overlapping phenotypes [13]. On the other hand, molecular tools can be helpful because they enable differentiation and significantly reduce diagnostics’ turnaround to a few hours. In the current scenario, nested PCR [14], PCR-RFLP (restriction fragment length polymorphism) [15], AFLP fingerprinting [9], multilocus microsatellite typing (MLMT) [16], DNA sequencing followed by phylogenetic analysis (e.g., ITS, *ARF*, *TUB1*, and *GP43*) [17,18], and whole-genome sequencing [19,20] are recommended for recognizing cryptic *Paracoccidioides* species. However, due to practical and technical challenges, such approaches are not frequently used in clinical laboratories.

Therefore, this study sought to develop a single-tube probe-based quantitative polymerase chain reaction (qPCR) to diagnose classic PCM due to members of the *P. brasiliensis* complex and *P. lutzii*. Our strategy uses one to three hydrolysis (TaqMan) probes with fluorescence resonance energy transfer (FRET) technology and a single pair of primers to detect *Paracoccidioides* DNA originating from isolated cultures, fresh specimens, or formalin-fixed paraffin-embedded tissue samples (FFPE). Moreover, our multiplex qPCR system can potentially boost diagnostic power in *Paracoccidioides*-affected regions, helping to reduce the time and costs without losing diagnostic sensitivity and specificity.

## 2. Materials and Methods

### 2.1. Ethics Approval

Approval was obtained from the ethics committee of the Federal University of São Paulo under protocol numbers 9771060120 and 3147220120.

### 2.2. Fungal Isolates and Culture Conditions

Seventy-seven *Paracoccidioides* isolates were obtained from the fungal collection of the Laboratory of Emerging Fungal Pathogens at the Federal University of São Paulo (UNIFESP), São Paulo, Brazil. All isolates were cultivated as slants on a Fava-Netto medium [21,22] at 37 °C in the yeast phase. *Paracoccidioides* isolates were previously identified at the species level using *TUB1*-RFLP [15] and AFLP markers [9]. Selected isolates represent various genotypes, origins, and clinical manifestations, and reference strains were used in all experiments (Supplementary Table S1).

### 2.3. DNA Extraction

Following the manufacturer’s instructions, DNA extraction and purification were performed on 10-day-old yeast colonies employing the Fast DNA kit methodology (MP Biomedicals, Vista, CA, USA) [23]. The NanoDrop 2000 spectrophotometer (Thermo Fisher Scientific, Waltham, MA, USA) was used to determine the concentration and purity of the DNA using the default setting of 1 OD (optical density) = 50 ng/µL double-stranded DNA at wavelengths of 260 and 280 nm. Only DNAs with ODs of 260/280 ratios between 1.8 and 2.0 were kept in this investigation. Before use, DNAs were diluted in nuclease-free water to a concentration of 50 ng/μL and stored at −20 °C. PCRs were conducted as a quality control procedure for *Paracoccidioides* DNA extraction using the ribosomal DNA operon-targeting universal primers ITS1 and ITS4 [24]. Samples that were successfully amplified were judged to be absent of inhibiting factors.

### 2.4. Primer and Probe Design and In Silico PCR

The rDNA sequences (18S, ITS1/2 + 5.8S, 28S, IGS) from 31 *Paracoccidioides* strains were used to develop genus-specific primer pairs and complex-specific probes (Supplementary Table S2) [20]. In addition, DNA sequences previously deposited in GenBank were retrieved to increase variability in the dataset [4,7,17,25,26]. The rDNA sequences were aligned using MAFFT software v. 7 [27] and manually curated in MEGA v7 [28] to prevent mispairing. The development of primers was based on the choice of short regions (18–25 nucleotides) containing parsimonious informative sites that were conserved at genus level and, whenever possible, divergent from other closely related Onygenales. Candidate sequences were evaluated using Primer3 [29] to calculate melting temperatures, %GC contents, dimer sequences, and mismatches. The Mfold software [30] evaluated putative secondary structures in candidate primers that would decrease amplification effectiveness. After all, DNA-based TaqMan probes (20–40 nucleotides) were designed to distinguish *P. lutzii* (species-specific probe), members of the *P. brasiliensis* complex (complex-specific probe), or *Paracoccidioides* species (genus-specific probe). Melting temperatures (Tm), annealing temperature, %GC contents, and mismatches in DNA-based TaqMan probes were calculated using the Primer Express software (Applied Biosystems, Carlsbad, CA, USA), considering MGB or QSY probes.

### 2.5. In Silico Validation of Primers and Probe Sequences

Primer-BLAST was used to test the specificity of the primers Paracoco-F and Paracoco-R and to conduct additional in silico PCR analysis [31]. To find unintentional, comparable targets, the stringency of primer specificity was tuned to detect sequences with at least 2 total mismatches, and at least 1 mismatch had to occur within the last 5 bps at the 3′ end. Targets with a single primer and one or more mismatches were eliminated. Following this, we mined the Primer-BLAST findings for sequences using the candidate probe sequences.

### 2.6. qPCR Optimization

The triplex-probe qPCRs were run in a final volume reaction of 20 μL using an Applied Biosystems StepOne Plus real-time PCR system (Applied Biosystems, Carlsbad, CA, USA). Reactions contained 2 μL of gDNA (25–50 ng/μL) as a template, 1.8 µL of sense primer, 0.6 µL antisense primer (10 pmol/μL; Integrated DNA Technologies, San Diego, CA, USA), 0.5 µL of each TaqMan probe (250 nM), 10 μL of TaqMan Genotyping Master Mix (2×) containing the passive internal reference dye ROX (Catalog No. 4371355; Applied Biosystems), and 4.1 μL of nuclease-free water. TaqMan probes (6000 pmol) were purchased from Thermo Fisher Scientific and labeled with FAM (518 nm), VIC (554 nm), or NED (575 nm) at the 5′ ends and with QSY quenchers or the non-fluorescent quencher-minor groove binder (NFQ-MGB) at their 3′-end. Primers and DNA-based FRET probe sequences are listed in Table 1. For the duplex-probe qPCR assay, the *P. brasiliensis* complex and *P. lutzii* TaqMan probes were added (0.5 µL each), and the reaction volume was set using nuclease-free water. A unique TaqMan probe (0.5 µL) was added for the singleplex-probe qPCR assay, and the reaction volume was adjusted using nuclease-free water. 

Standard thermal cycling conditions were performed as follows: (1) an initial step of 2 min at 50 °C and 10 min at 95 °C, followed by (2) 40 cycles of 15 s at 95 °C (denature) and 1 min at 60 °C (anneal/extend). The fluorescent signal collection was allowed at the extension step. All qPCR experiments were run with negative controls using nuclease-free water and positive controls (Pb18 and Pb01 gDNA). Thus, positive and negative results were considered valid for a given experiment only after the absence of signal detection for negative controls, along with specific detection for positive controls.

Amplicons generated with primers Paracoco-F and Paracoco-R were purified employing the Wizard SV Gel and PCR Clean-Up System (Promega, Madison, WI, USA). To improve the quality of the sequenced data (Phred ≥ 30), the fungal ITS2 amplicons were control sequenced in two reactions. A BigDye Terminator v3.1 Cycle Sequencing Kit from Applied Biosystems was used for the Sanger sequencing procedures, and a SeqStudio Genetic Analyzer System was used to identify the sequences (Applied Biosystems).

### 2.7. Determining Assay Specificity In Vitro

The specificity of the TaqMan probes was validated using a triplex-probe qPCR assay along with *Paracoccidioides* species DNA (n = 77; Supplementary Table S1). Moreover, to investigate our assays’ cross-reactivity with other medically relevant fungi, we used a set of non-target DNAs from agents of superficial, subcutaneous, and systemic mycoses in humans and animals (Supplementary Table S3). Human DNA isolated from A549 cell samples [32] and BALB/c DNA isolated from mouse lungs [33] were used to investigate if our assay cross-reacts with host DNA. A multiplex PCR-based quality-control assay using primers targeting nonoverlapping sites in the GAPDH gene (chr12) of the human genome was used to check the quality of our A549 cell DNA extraction [34], and samples that returned 100, 200, 300, and 400 bp amplicons were deemed to be free of PCR inhibitors [33]. Murine DNA quality was evaluated by conventional PCR using the primers targeting the β-actin gene in the BALB/c genome, as described by Pahl et al. [35], and samples with positive amplification were regarded to be free of PCR inhibitors.

### 2.8. Determining Assay Sensitivity

A 10-fold serial dilution regression analysis was used to ascertain the detection limits, beginning with 100 ng/µL and finishing with 0.01 fg/µL of *Paracoccidioides* gDNA. Likewise, we performed a 10-fold serial dilution regression analysis of ITS2 amplicons (amplified using primers Paracoco-F and Paracoco-R), beginning with 3 × 10^6^ copies per tube and finishing with 3 copies per tube. As previously described, each probe’s limit of detection (LoD) was observed under singleplex-, duplex-, and triplex-probe settings [36]. All standard curves were performed in triplicate.

### 2.9. Target Template Competition

Serial 10-fold dilutions were tested to establish the performance of our triplex-probe qPCR assay to detect a mixed infection of any member of the *P. brasiliensis* complex and *P. lutzii*, as previously described by Della Terra et al. [36]. In the first experiment, increasing concentrations of *P. brasiliensis* complex (Pb18) and *P. lutzii* (Pb01) were included in the qPCR assays. In a second experiment, to evaluate the template competition, the highest tested concentration of the *P. brasiliensis* gDNA (i.e., 100 ng) was combined with the lowest tested amount of *P. lutzii* gDNA (i.e., 0.01 fg) and added to a triplex-probe qPCR assay. The permutations were carried out until the maximum amount of the opposing species’ DNA (i.e., 100 ng of *P. lutzii* DNA) was combined with the first species’ lowest tested DNA amount (i.e., 0.01 fg of *P. brasiliensis* DNA). All reactions were run in the presence of 50 ng of human DNA as a spike. In addition, two negative controls using nuclease-free water spiked with or without A549 DNA were run for each qPCR assay.

### 2.10. Detection of Paracoccidioides DNA from Formalin-Fixed, Paraffin-Embedded (FFPE) Tissue and Other Biological Samples

For the clinical validation assay, sixteen formalin-fixed, paraffin-embedded (FFPE) tissue samples from the tibia (n = 2), duodenum (n = 1), lungs (n = 2), lymph node (n = 5), oral mucosa (n = 2), kidney (n = 1), and skin (n = 3) were obtained from 12 Brazilian patients (14–66 years old; Supplementary Table S4). Histopathology confirmed paracoccidioidomycosis in each sample. Eighteen FFPE tissue samples from patients with other diseases (non-PCM) were included as negative controls: human sporotrichosis, n = 1; human leishmaniasis, n = 10; tuberculosis, n = 6; and histoplasmosis, n = 1. DNA was extracted from three 5 µm thick sections of each FFPE using the QIAamp DNA FFPE Tissue Kit (Qiagen, Hilden, Germany), observing the guidelines provided by the manufacturer [37,38]. All FFPE samples were taken in strict accordance with legal requirements and best-practice standards [39,40,41]. The FFPE isolated DNA was resuspended in 70 µL of elution buffer [38,42], and DNA quality was evaluated by running a quadruplex assay targeting the GAPDH gene (chr12) [34]. Those FFPE DNAs that produced at least three fragments (e.g., 100, 200, and 300 bp) were considered non-refractory to further qPCR analyses. For the triplex-probe qPCR assay, we added 5 µL of FFPE DNA. The standard qPCR thermal cycling conditions were used.

In addition, nine fresh clinical samples (sputum = 1; fresh tissue biopsy = 3; bronchoalveolar lavage = 4; cerebrospinal fluid = 1) were taken from nine patients (30–73 years old) in Brazil (Supplementary Table S4). During the direct mycological examination, PCM was proved in all nine patients by laboratory demonstration of pathognomonic “steering wheel” budding yeast cells. DNA extraction and purification were performed using the Fast DNA kit procedure and the CLS-TS component (MP Biomedicals, Vista, CA, USA), following the manufacturer’s directions [43]. DNA concentration and purity were assessed as described above for FFPE samples. A volume of 5 µL of clinical samples’ DNA was investigated using the triplex-probe qPCR assay.

### 2.11. Detection of Paracoccidioides DNA from Spiked Soil Samples

Attempts were made to extract DNA from artificially contaminated soil (spiked) to establish the assay’s feasibility for sensitive and specific detection of *Paracoccidioides* DNA. An aliquot of 50–100 mg soil samples was used for DNA extraction employing the Fast DNA kit procedure and the CLS-VF/PPS component (MP Biomedicals, Vista, CA, USA), following the manufacturer’s directions [43]. Subsequently, we used 2 μL of the soil DNA (100 ng/μL) along with a 10-fold serial dilution of the *Paracoccidioides* DNA (spiked reactions), beginning with 100 ng/μL and finishing with 0.01 fg/μL. Non-spiked reactions were included as negative controls [33]. The triplex-probe qPCR assay was run as previously specified using the standard cycling conditions.

### 2.12. Statistical Analysis

Performance indicators such as sensitivity, specificity, positive predictive value (PPV), and negative predictive value (NPV) were calculated for the triplex-probe qPCR assay. To evaluate the usefulness of various tests for diagnosing PCM, we employed the receiver operating characteristic (ROC) curves and the area under the curves (AUC) to assess the diagnostic accuracy of our assay. In addition, we determined the kappa statistic and its 95% confidence interval (CI) to establish the degree of concordance between the results from our triplex-probe qPCR assay and those from the conventional duplex PCR previously reported by our group [33]. The kappa rates were interpreted as follows: values between 0.00–0.20, poor agreement; 0.21–0.40, fair agreement; 0.41–0.60, moderate agreement; 0.61–0.80, good agreement; and 0.81–1.00, perfect agreement [44]. All analyses were carried out using the statistical program MedCalc (v.20.114), and if the *p*-values were 0.05 or lower, the results were deemed as significant.

## 3. Results

### 3.1. Primers and Probes Design

**Figure 1:**
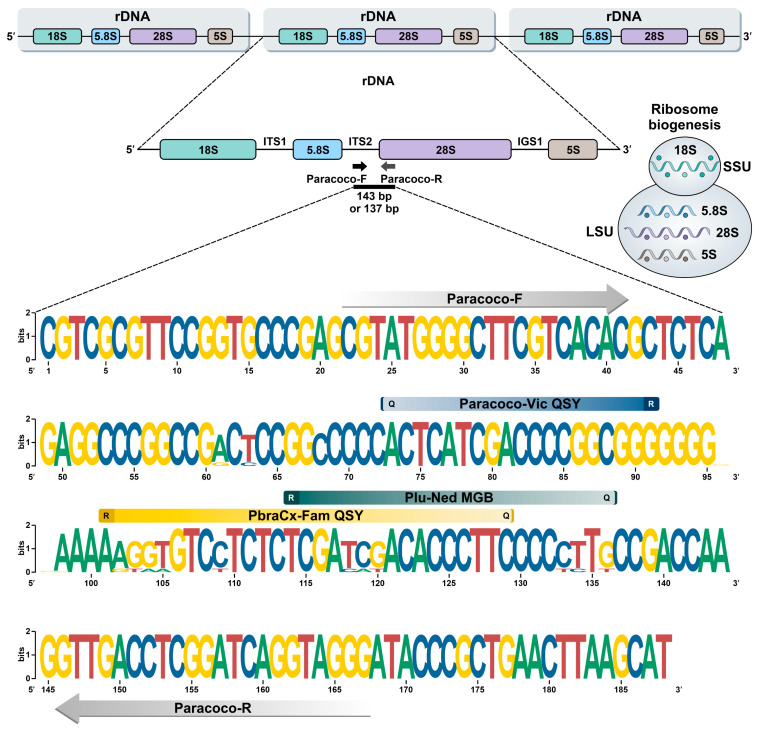
Graphic representation using WebLogo of polymorphism patterns within the alignment of 31 target sequences of members of the *P. brasiliensis* complex and *P. lutzii* (see Supplementary Table S2). Paracoco-F and Paracoco-R primers recognize a highly conserved region for *Paracoccidioides* species with no mismatches. Parsimony-informative sites in the ITS2 region uncovered candidate areas for DNA-based FRET probes design, i.e., PbraCx-Fam, Plu-Ned, and Paracoco-Vic. R: reporter; Q: quencher.

The full dataset comprised 4073 bp long rDNA sequences aligned with MAFFT software and partially covered the 18S, ITS1/2 + 5.8S, and 28S, including 4026 invariable characters (98.84%), 39 variable parsimony-informative sites (0.95%), and 6 singletons. Primers Paracoco-F and Paracoco-R targeted the conserved region (invariable characters) of ITS2 and 28S, respectively. In silico PCR using the primers Paracoco-F and Paracoco-R generated a 143 bp fragment for members of the *P. brasiliensis* complex and a 137 bp amplicon for *P. lutzii*. DNA-based FRET probes lying in the ITS2 were designed to deliver a generic identification (Paracoco-Vic probe) or to recognize members of the *P. brasiliensis* complex (PbraCx-Fam probe) and *P. lutzii* (Plu-Ned probe) in a single-round reaction (Figure 1; Table 1). To validate our results, we performed an in silico analysis with the primer BLAST software, and no target templates were found in the nucleotide collection (nt) database for species different from *Paracoccidioides* (Supplementary Table S5). Moreover, our analyses revealed that probe-binding regions were polymorphic among other closely related Onygenales, such as *P. cetii* and *P. loboi*, preventing unspecific recognition (Supplementary Figure S1). Therefore, the assay accuracy relies on using the TaqMan probes. 

### 3.2. qPCR Standardization

Our initial attempts to standardize the triplex-probe qPCR assay using a 3 × 3 matrix (100 nM, 300 nM, and 900 nM) indicated that the forward primer at a final concentration of 900 nM, reverse primer at a final concentration of 300 nM, and probes at a final concentration of 250 nM are the ideal combination for *P. brasiliensis* (Cq = 20.25 ± 0.09) and *P. lutzii* DNAs (Cq = 18.28 ± 0.71) as templates.

### 3.3. qPCR Efficiency

**Figure 2:**
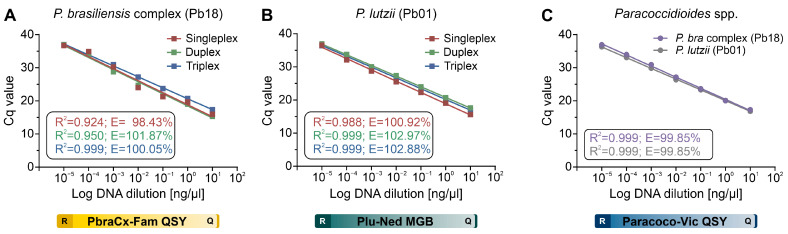
Standard curves were constructed for (**A**) *P. brasiliensis* probe (Pb18 DNA, PbraCx-Fam), (**B**) *P. lutzii* probe (Pb01 DNA, Plu-Ned), and (**C**) *Paracoccidioides* species probe (Pb18 or Pb01 DNAs, Paracoco-Vic) under a singleplex-probe (red line), a duplex-probe (green line), or a triplex-probe qPCR assay (blue line), showing comparable efficiencies and that the qPCR settings are multiplex-compatible. The results are typical of two separate experiments. R: reporter; Q: quencher.

The 10-fold serial dilution regression analysis under a singleplex-, duplex-, and triplex-probe condition with their respective *Paracoccidioides* species is shown in Figure 2. For the gDNA, it was observed that the amplification covers all dilutions of the qPCR efficiency at good Cq values for the *P. brasiliensis* probe (singleplex-probe Cq = 15.96–36.70; duplex-probe Cq = 15.28–36.99; triplex-probe Cq = 17.37–36.97), *P. lutzii* probe (singleplex-probe Cq = 15.62–36.56; duplex-probe Cq = 17.66–37.01; triplex-probe Cq = 16.92–36.42), and *Paracoccidioides* species probe (*P. brasiliensis* DNA Cq = 17.33–36.95 and *P. lutzii* DNA Cq = 16.86–36.31).

All standard curves revealed a strong linear correlation (R^2^ = 0.92–0.99) for all tested probes under any plex-probe condition. Our qPCR assay showed excellent efficiency and reproducibility among replicates in the *P. brasiliensis* probe (singleplex-probe E = 98.43%; duplex-probe E = 101.87%; triplex-probe E = 100.05%), *P. lutzii* probe (singleplex-probe E = 100.92%; duplex-probe E = 102.97%; triplex-probe E = 102.88%), and *Paracoccidioides* species probe (*P. brasiliensis* DNA E = 99.85% and *P. lutzii* DNA E = 99.85%). As a result, qPCR efficiency analysis revealed that the primer-probe combinations were consistent with formats ranging from single- to multiplexing.

### 3.4. Analytical Sensitivity (LoD)

**Figure 3:**
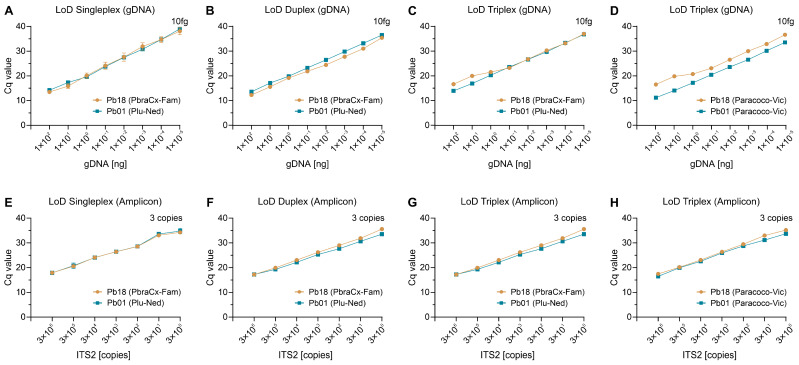
Assessment of analytical sensitivity of a singleplex- (**A**,**E**), duplex- (**B**,**F**), and triplex-probe qPCR assay (**C**,**D**,**G**,**H**). (**A**–**D**) For *P. brasiliensis* (Pb18) and *P. lutzii* (Pb01), the lower detection limit of gDNA was 10 fg, independent of the format. (**E**,**F**) For all species, the lower limit of detection of the ITS2 amplicon (amplified with primers Paracoco-F and Paracoco-R) was three copies. For each format, the detected probe appears in parentheses in the legend. The results are typical of two separate experiments.

By testing amplification using 10-fold serial gDNA dilutions, we could estimate our assay’s analytical sensitivity, and regardless of the test set, the gDNA detection limit for all targets was 10 fg (singleplex, Figure 3A; duplex, Figure 3B; or triplex, Figure 3C,D). To perform qPCR analysis with greater accuracy, we assessed the analytical sensitivity using the ITS2 amplicon template generated with primers Paracoco-F and Paracoco-R, and under any plex-probe scenarios, three copies of each target were the lowest concentration found (Figure 3E–H).

### 3.5. Analytical Specificity

**Figure 4:**
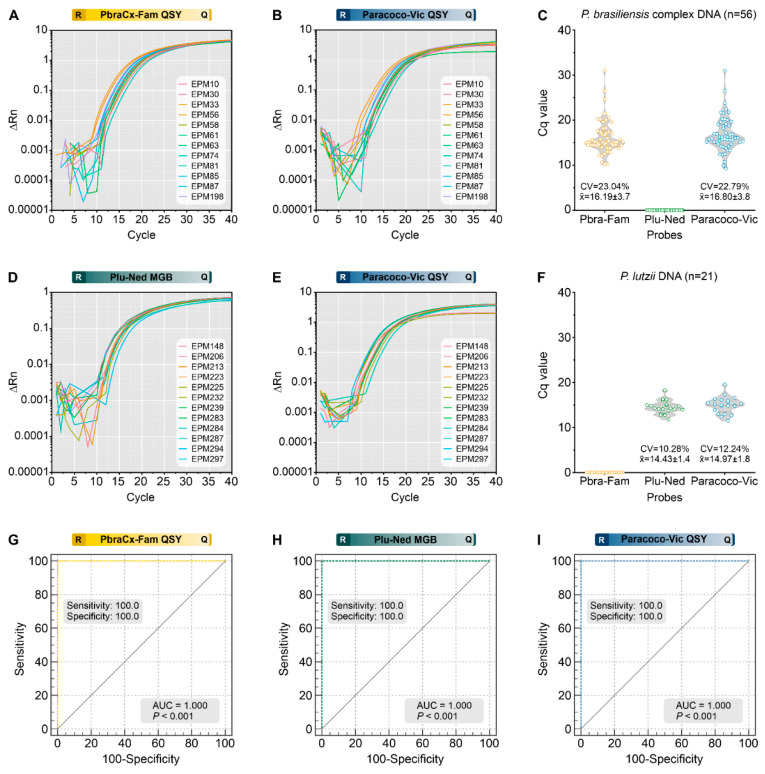
Assessment of analytical specificity of a triplex-probe qPCR assay for *Paracoccidioides* species. The representative amplification plot for PbraCx-Fam probe (**A**) or Paracoco-Vic probe (**B**) and the DNA of members of the *P. brasiliensis* complex; (**C**) Scatter plot of Cq values (y-axis) from a group of 56 *P. brasiliensis* complex DNA, including *P. brasiliensis s. str.* (n = 42), *P. americana* (n = 6), *P. restrepiensis* (n = 5), and *P. venezuelensis* (n = 3). The representative amplification plot for Plu-Ned probe (**D**) or Paracoco-Vic probe (**E**) and the DNA of *P. lutzii*; (**F**) Scatter plot of Cq values (y-axis) from a group of 21 *P. lutzii* DNA. (**G**–**I**) ROC analyses were calculated for 56 isolates of *P. brasiliensis* complex, 21 isolates of *P. lutzii*, and 28 isolates of other pathogenic fungi (Supplementary Table S3), revealing the utility of our qPCR test (AUC = 1.000, 95% CI = 0.964–1.000, *p* < 0.0001) in identifying *Paracoccidioides* species. (**G**) ROC analysis for the PbraCx-Fam probe reached 100% specificity and sensitivity as only 56 isolates of *P. brasiliensis* complex were positive. (**H**) ROC analysis for the Plu-Ned probe revealed 100% specificity and sensitivity as only 21 isolates of *P. lutzii* were positive. (**I**) ROC analysis for the Paracoco-Vic probe showed 100% specificity and sensitivity as 77 *Paracoccidioides* spp. isolates were positive. CV: coefficient of variation. The results are typical of two separate experiments. R: reporter; Q: quencher.

A panel of 77 *Paracoccidioides* DNA samples (Supplementary Table S1) was used to assess assay specificity in this study, and amplification plots are depicted in Figure 4A (PbraCx-Fam), Figure 4B (Paracoco-Vic), Figure 4D (Plu-Ned), and Figure 4E (Paracoco-Vic). The qPCR resulted in specific amplification products from all members of the *P. brasiliensis* complex (n = 56; PbraCx-Fam Cq = 10.15–30.97; PbraCx-Fam Median Cq = 15.13; Paracoco-Vic Cq = 9.26–30.97; Paracoco-Vic Median Cq = 16.02; Figure 4C), and *P. lutzii* (n = 21; Cq = 11.75–18.28; Median Cq = 14.25; Paracoco-Vic Cq = 11.53–19.59; Paracoco-Vic Median Cq = 14.95; Figure 4F). Non-specific amplification products were absent. Our triplex-probe qPCR assay was in full conformity (100%) with the identifications achieved with molecular *TUB1*-RFLP [15] and AFLP markers [9] (kappa = 1.0; 95% CI = 1.0–1.0; perfect agreement) performed previously at our laboratory, reinforcing the value of differentiating the critical agents of classic PCM. Thus, no cross-reactivity was observed.

Additionally, we used DNAs from 25 medically relevant fungi other than *Paracoccidioides* to confirm the specificity of our assay, and no measurable fluorescent signal was observed. Moreover, the ability of our triplex-probe assay to reveal *Paracoccidioides* in the presence of host DNA was further supported by the absence of cross-reactivity with human (A549 cell line) or murine DNAs (BALB/c). This finding corroborates the high specificity projected by the BLASTn-search assessments. Finally, AUC investigation provided a clear indication of the assay’s usefulness in discriminating between members of the *P. brasiliensis* complex and *P. lutzii* (Figure 4G,H) or by achieving genus-level identification (AUC = 1.000, 95% CI = 0.964–1.000, *p* < 0.0001) (Figure 4I).

### 3.6. Target Template Competition

**Figure 5:**
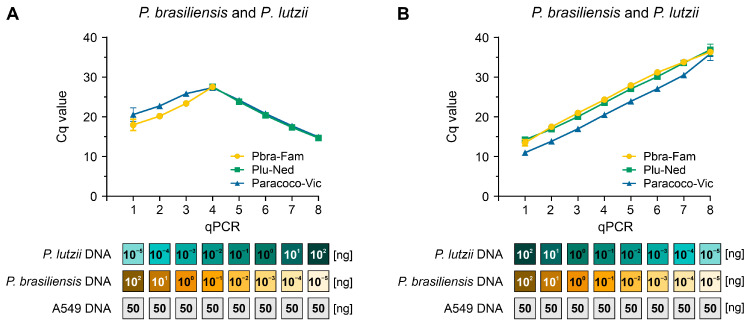
Assessment of target/template competition. (**A**) Using a triplex-probe qPCR assay, we combined the gDNA of *P. brasiliensis* (Pb18) and *P. lutzii* (Pb01) in a manner that the highest DNA concentration for the first species in the curve was mixed with the lowest concentration in the opposite species’ curve. (**B**) *P. brasiliensis* (Pb18) and *P. lutzii* (Pb01) gDNA were added to the triplex-probe qPCR assay in equimolar amounts. All qPCRs were run in the presence of 50 ng human DNA. The results are typical of two separate experiments.

In a hypothetical setting where we had two different targets (for example, *P. brasiliensis* and *P. lutzii*) in the same qPCR with varying quantities of gDNA, we assessed the viability of our assay to recognize both targets. In all simulations evaluated, we observed signals for a single *Paracoccidioides* species along with the signal for the genus probe. We detected both species in those reactions with up to a 10-fold difference in DNA concentration (for example, 10^−2^ ng of *P. brasiliensis* DNA and 10^−1^ ng of *P. lutzii* DNA) (Figure 5A). On the other hand, equimolar concentrations of the different targets used in a 10-fold serial dilution did not affect the templates’ capacity to be detected by our triplex-probe assay (Figure 5B). Therefore, this suggests that we could detect PCM co-infections, but a higher concentration of one of the targets under a triplex-probe setting will favor its detection over the less abundant target.

### 3.7. Detection of Paracoccidioides DNA from Clinical Samples

**Figure 6:**
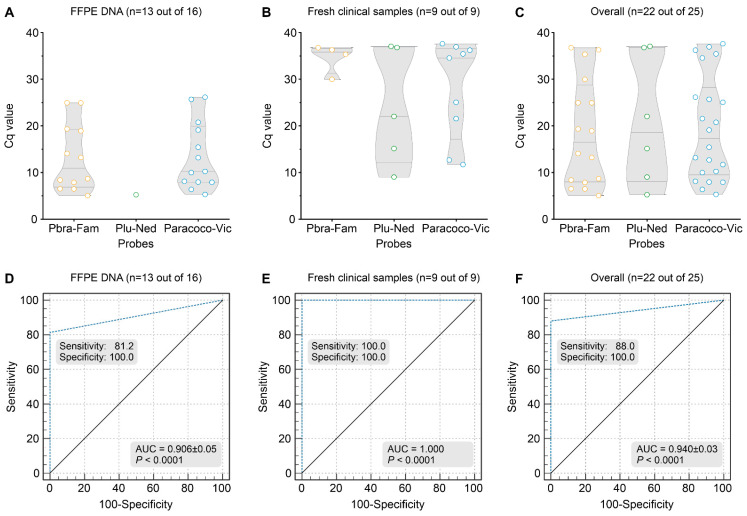
Summary of FFPE tissues (n = 16) or miscellaneous fresh clinical samples (n = 9), including sputum, pus, tissue biopsy, bronchoalveolar lavage, and cerebrospinal fluid, employed in our triplex-probe qPCR. (**A**) Scatter plot of Cq values (y-axis) from a group of 16 DNAs revealed that 13 specimens were positive for the Paracoco-Vic probe (81.2%; Mean Cq = 10.25), with 12 *P. brasiliensis* complex (PbraCx-Fam) and a single *P. lutzii* detected (Plu-Ned probe). (**B**) Scatter plot of Cq values (y-axis) from a group of nine DNAs revealed that nine specimens were positive for the Paracoco-Vic probe (100%; Mean Cq = 27.98), with four *P. brasiliensis* complex (PbraCx-Fam) and five *P. lutzii* detected (Plu-Ned probe). (**C**) Scatter plot of Cq values (y-axis) from overall samples (FFPE and fresh clinical samples). (**D**) ROC analysis for the triplex-probe qPCR reached 100% specificity, and 81.2% sensitivity as 13 FFPE samples were positive and only 3 samples were false-negative (AUC = 0.906 ± 0.05; *p* < 0.0001, 95% CI = 68.319–94.313, Youden index J = 0.8125, NPV = 85.67%, PPV = 100%). The 18 non-PCM samples remained negative. (**E**) ROC analysis for the triplex-probe qPCR reached 100% specificity and sensitivity as 9 fresh clinical samples were positive, and the 18 non-PCM samples remained negative (AUC = 1.000; *p* < 0.0001, 95% CI = 0.872–1.000, Youden index J = 1.000, NPV = 100%, PPV = 100%). (**F**) ROC analysis for the triplex-probe qPCR reached 100% specificity, and 88% sensitivity as 22 specimens were positive (13 FFPE samples and 9 fresh clinical samples) and only 3 samples were false-negative (AUC = 0.940 ± 0.03; *p* < 0.0001, 95% CI = 0.823–0.989, Youden index J = 0.8800, NPV = 85.7%, PPV = 100%). The 18 non-PCM samples remained negative. The results are typical of two separate experiments.

FFPE tissue biopsies from patients with confirmed PCM infection (n = 21) and patients with other disorders (n = 18) were used to measure our assay’s ability to detect *Paracoccidioides* DNA (Supplementary Figure S2). Of the 39 FFPE samples included in the study, 34 tested positive for at least three bands using the GAPDH control, and five samples from the PCM group were removed due to the low quality of FFPE DNA (i.e., no amplification of GAPDH control; Supplementary Figure S2). From the 16 GAPDH-positive samples of the PCM group, 12 specimens were positive for the *P. brasiliensis* complex probe (Cq = 5.05–24.96; Median Cq = 10.96) and a single specimen for the *P. lutzii* probe (Plu-Ned Cq = 5.28). All thirteen samples were positive for the *Paracoccidioides* probe (Cq = 5.30–26.17; Median Cq = 10.25, Figure 6A). Three samples remained negative under the triplex-probe assay after two rounds of qPCR. For the non-PCM group, the 18 samples tested negative for the 3 probes evaluated (Supplementary Figure S2). Sensitivity (81.25%), specificity (100%), the area under the ROC curve (0.906 ± 0.0504; *p* < 0.0001), Youden index (0.8125), and negative (NPV = 85.67%) and positive predictive values (PPV = 100%) of qPCR were calculated using the results obtained with 16 FFPE samples of PCM patients and 18 FFPE samples of other diseases (95% CI = 68.319–94.313) (Figure 6D). 

All fresh tissue samples tested positive for at least three bands using the GAPDH control (Supplementary Figure S2). From the nine samples of the PCM group, four samples were positive for the *P. brasiliensis* complex probe (Cq = 29.99–36.78; Median Cq = 35.84) and five samples for *P. lutzii* probe (Cq = 9.03–37.07; Median Cq = 22.05). All nine samples were positive for the *Paracoccidioides* probe (Cq = 11.70–37.62; Median Cq = 34.56, Figure 6B). For the non-PCM group, the 18 samples tested negative for the 3 probes evaluated (Supplementary Figure S2). Sensitivity (100%), specificity (100%), the area under the ROC curve (1.000; *p* < 0.0001), Youden index (1.000), and negative (100%) and positive predictive values (100%) of qPCR were calculated using the results obtained with 9 clinical specimens of diseased patients and 18 samples of other diseases (95% CI = 0.872–1.000) (Figure 6E). 

Using kappa statistics, a comparison between qPCR and a conventional duplex PCR result for FFPE or fresh clinical samples revealed significant agreement (i.e., 13 detectable and 3 undetectable for FFPE samples, and 9 detectable for fresh clinical samples; kappa = 1.0; see Supplementary Figure S2).

### 3.8. Detection of Paracoccidioides DNA from Spiked Soil Samples

**Figure 7:**
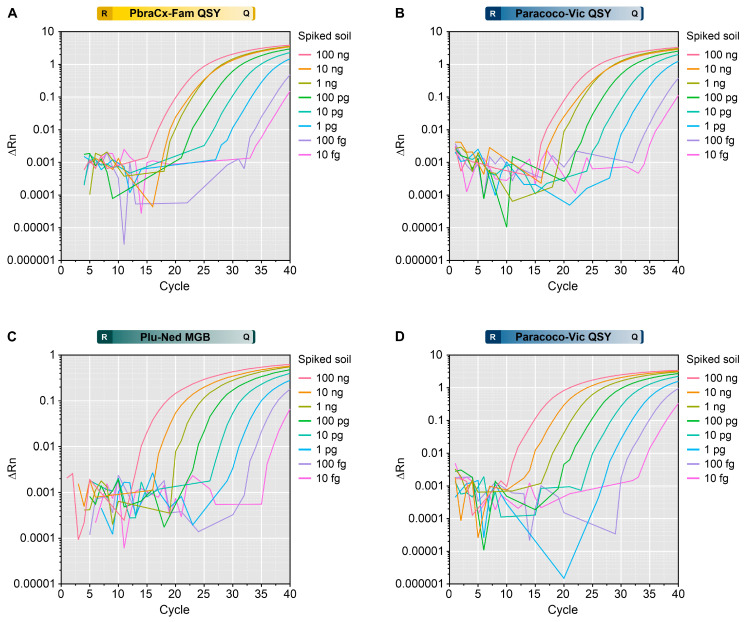
Assessment of a triplex-probe qPCR to detect (**A**,**B**) *P. brasiliensis* or (**C**,**D**) *P. lutzii* DNA in the spiked soil. The amplification plot for the PbraCx-Fam probe (**A**) or Paracoco-Vic probe (**B**) and the DNA from Pb18. The amplification plot for the Plu-Ned probe (**C**) or Paracoco-Vic probe (**D**) and the DNA from Pb01. Different colors of amplification curves represent samples from soil spiked with different inputs of *Paracoccidioides* DNA (100 ng–10 fg). ΔRn relates to the magnitude of the normalized reporter value (fluorescence signal) used to construct the standard curve. The results are typical of two separate experiments. R: reporter; Q: quencher.

The sensitivity of our assay to detect *Paracoccidioides* DNA was evaluated using soil samples spiked with *P. brasiliensis* or *P. lutzii* DNAs. The amplification curves indicated positive signals from spiked soil material, and the Cq values correlated with the input amount of DNA. The minimum detection level for *Paracoccidioides* in soil for all probes was 10 fg of gDNA (Figure 7). There was no amplification beyond the cycle threshold in the negative control samples, which included sterile, non-spiked soil (no false positives). This suggests that our qPCR can specifically detect DNA from *Paracoccidioides*, demonstrating its high specificity and potential use in environmental research.

## 4. Discussion

Infections caused by fungi threaten human health worldwide, and presumably, millions of people live with severe fungal infections [45,46,47]. As observed for PCM, a neglected mycosis with significant morbidity and mortality in Latin America, many lives could be preserved by applying quick and accurate diagnosis [48]. Despite PCM clinical relevance, diagnostics tools have been challenging, mainly because classical techniques such as culturing, histopathology, and phenotypic methods are laborious and time-consuming. We developed a triplex-probe assay targeting the *Paracoccidioides* rDNA region (ITS2/28S) to overcome this problem of detecting DNA from isolated cultures to clinical samples. Our qPCR assay is an important tool for understanding epidemiological patterns, mapping the affected areas, and guiding early treatment of patients affected by the disease [49]. 

Our multiplex qPCR presents the following improvements: (i) it simplifies the differentiation of *P. lutzii* from other members of the *P. brasiliensis* complex; (ii) it recognizes the main culprits of classic PCM in a one-tube reaction, thus saving qPCR components and the steps required to distinguish *Paracoccidioides*; (iii) it is more sensitive than isolation in culture [5] and is at least 100 times more sensitive than our previous duplex PCR [33]; (iv) it is ideal for detecting *Paracoccidioides* in fresh specimens or FFPE tissue samples and exhibits no cross-reaction with human or murine DNA; (v) it is suitable for the detection of *Paracoccidioides* in environmental samples; and (vi) it is straightforward to interpret (Supplementary Figure S3), expanding the offer of a highly effective assay to distant areas in South America, a critical bottleneck in a neglected tropical disease scenario.

The species boundaries in *Paracoccidioides* impact the development of diagnostic tests [49]. Thus, despite good PCR-based tools available in the literature [4,10,11,50,51,52,53], our assay has the advantage of covering the recent taxonomic developments in the genus [1,4,17,18,54]. The *P. brasiliensis* complex comprises cryptic species accumulating genetic differences without adding significant clinical relevance [1]. Recognizing members of the *P. brasiliensis* complex down to the species level requires laborious approaches such as MLSA schemes [1,18] or whole-genome sequencing [19,20,55], a scenario distant from the reality of clinical laboratories in South America, where PCM is endemic [56]. 

From a serological perspective, it is not uncommon to find patients with negative serological results for the B-339 antigen in double immunodiffusion assay but positive in direct mycological examination [5,57,58,59,60,61]. In these paradoxical cases, identifying *P. lutzii* may be essential to recognizing false negative cases in serology and mapping the areas where regional antigens from *P. lutzii* are needed. To our knowledge, qPCR assays that allow the identification of all agents embedded in the *P. brasiliensis* complex are inexistent due to the technical complexity of multiplexing more than three DNA-based FRET probes labeled with fluorescent dyes in addition to the unreasonable cost generated by this experimental design. Moreover, judging from the clinical perspective, speciating members of the *P. brasiliensis* complex has no impact on patient management [5,6,8,62,63,64]. Therefore, differentiating between members of the *P. brasiliensis* complex and *P. lutzii* seems to be, at this moment, the safest and most cost-effective strategy for diagnosing classic PCM [1,33,49].

Our qPCR test can be used in different formats according to the needs and budget of each laboratory. For example, the singleplex assay employing only the Paracoco-Vic probe grants the genus-level diagnosis of classic PCM (Supplementary Figure S3). The duplex assay with the PbraCx-Fam and Plu-Ned probes allows the diagnosis of mixed infections by any of the members of the *P. brasiliensis* complex and *P. lutzii* (Supplementary Figure S3). However, similar to our previous conventional duplex PCR assay targeting the GP43 gene [33], here it is impossible to detect mixed infections by any two members of the *P. brasiliensis* complex (e.g., *P. americana* and *P. brasiliensis s. str*., or *P. restrepiensis* and *P. venezuelensis*) since a single positive signal would be detected (i.e., PbraCx-Fam). From our perspective, the triplex assay is the most attractive option because it offers a double confirmation: first, generic (*Paracoccidioides* spp.) and second, specific, which allows a significant coverage of the diversity of *Paracoccidioides*, bringing more confidence to the assay (Supplementary Figure S3).

The high sensitivity and specificity of our qPCR assay results from combining accurate genome-based design [20,55] and using fluorescently-labeled TaqMan probes. As for efficiency, we showed great linearity values (R^2^ nearly 1.0) and excellent slopes (~−3.33) for all curves. The amplification scale ranges from 1.0 (no amplification) to 2.0 (perfect exponential duplication), with scales ranging between 1.8 and 2.0 accepted as good [65], which is the case of all amplification results we had with a single-to-multiplex assay. These results reinforce that the presence of more than one probe in a single-round reaction does not reduce the efficiency of our assay. The number of isolates used here allowed us to verify that the AUC coincided with the total area of the ROC curve, and we assumed nearly 100% sensitivity and specificity in any format employed. The analytical sensitivity for gDNA was 10 fg/μL for gDNA, and using the ITS2 amplicon, we could detect up to three copies, according to Poisson’s distribution probability [6,66].

Sputum and tissue biopsies, including FFPE tissues, are the main clinical specimens used to diagnose classic PCM. A great performance of our test was observed with clinical samples, reducing the need for isolation of *Paracoccidioides*, thus saving critical time. Overall, the application of PCR-based diagnostics shows that the test might work better on sputum than in blood samples [50], whereas the tissue detection would have no problem if the available *Paracoccidioides* species’ DNA is greater than 10 fg [67], which is in agreement with our results. Therefore, we recommend detecting *Paracoccidioides* DNA in clinical routine and emphasize that the major feature of maintaining diagnostic accuracy is acquiring an excellent clinical specimen free of PCR inhibitors [49]. Moreover, judging from the retrospective nature of our study, we report a significant occurrence of *P. lutzii* as the etiological agent in areas remote from the epicenter of *P. lutzii*-mycosis in Central-West Brazil, confirming that *Paracoccidioides* species are pathogens on the move and epidemiological trends are not fully elucidated [9,15,68,69,70].

FFPE samples have been effectively applied in DNA sequencing and qPCR investigations [38,42], but this success is affected by several pre- and postprocessing issues. For example, downstream molecular analyses may be impacted by the nature and quantity of tissue, the fixative buffer employed for tissue conservation, the extent of fixation, the age of the paraffin block, and storage conditions, as well as the cross-links, the length of the chosen DNA segment to be amplified, and copy number variation [39,40,41]. We included FFPE specimens preserved for a decade (2010–2020) that underwent rigorous quality control by running a quadruplex assay targeting the human GAPDH gene [34]. A remarkable positivity was reported (81.2%) for our triplex-probe qPCR assay, underlining the potential for retrospective studies in FFPE tissue collections in Latin America. Moreover, the odds of detection were improved by choosing a target in the genic and spacer regions of the ribosomal cistron with significant copy number variation in *Paracoccidioides* genomes. Considering the natural fragmentation of DNA during the processing of FFPE tissue (cross-links), the smaller the size of the amplicon used for qPCR analysis, the greater the chance of detecting the specific target, and we report great success in amplifying a fragment of 137–143 bp in FFPE tissues.

A major pitfall of our study is the absence of clinical samples from patients with PCM loboi or cetaceans with PCM ceti [12,26,71], which is partially justified due to the unculturable nature of the etiological agents and the rarity of these infections compared with classic PCM [1,8]. A BLAST search using the Primer-BLAST tool and the sequences of Paracoco-F and Paracoco-R primers or probes did not retrieve any amplicon related to *P. loboi*, which could be associated with the scarcity of *P. loboi* rDNA sequences from public databases [7]. Judging from *P. cetii* sequences (GenBank accession numbers: MW566081–MW566084), a mutation in the forward primer and along the PbraCx-Fam (1 mutation out of 29; 3.44%) and Plu-Ned (10 mutations out of 18; 55.55%) probes binding regions may prevent recognition using our assay. The *Paracoccidioides* probe (Paracoco-Vic), on the other hand, had a 100% similarity with *P. cetii*, but not *P. loboi* (13 mutations out of 19; 68.42%). This strongly suggests that none of the probe and primer combinations would recognize *P. cetii* and *P. loboi* (Supplementary Figure S1).

Ecological studies on PCM present an important bottleneck due to the insufficient detection capacity of *Paracoccidioides* in its natural habitat, either by employing classical methods such as fungal isolation or molecular tools based on DNA detection [51,72,73,74,75,76,77]. Here, we demonstrated that our triplex-probe qPCR has the potential to identify *Paracoccidioides* in ecological studies using soil samples, the typical environmental sources of propagules [10,51,53,74,75,78]. Isolation of *Paracoccidioides* species from the environment is infrequently reported in endemic areas [76,77,79,80], which could be associated with the low concentration and seasonality of *Paracoccidioides* propagules in the soil or the fastidious development of *Paracoccidioides* species in nutrient-rich culture media compared to the rapidly growing Ascomycetes that lead soil diversity and abundance globally [81,82]. Recently, Arantes et al., using nested PCR and in situ hybridization methods, demonstrated that soil and aerosol samples were positive for *Paracoccidioides* spp. DNA, revealing the ordinary distribution of these microorganisms in the Brazilian territory [78]. Likewise, our qPCR assay employing spiked samples revealed the potential to gain insights into the distributions of *Paracoccidioides* and the conditions that boost their occurrence in the environment.

## 5. Conclusions

Diagnostics is a vital component of any successful public health policy, and the development of fast, reliable, and reasonably priced assays is critical for tailoring proper therapeutical strategies against PCM. Thus, the approach presented here simplifies laboratory diagnosis without delving into speciation processes, for which molecular markers are not yet defined and are still nebulous for routine use [1]. Our test fits current diagnostics necessities as it considerably reduces the turnaround time for fungal identification, dismisses the need for gel electrophoresis and downstream analyses, and reduces amplicon contamination and the manipulation of samples and cultures, which is a cost-effective choice for a neglected tropical disease associated with poverty. Moreover, our assay allows us to investigate eventual co-infections, which might be less rare than previously thought [33,42]. All these features are desirable to improve fungal diagnostic capacity and tackle the spread of classic PCM in a vast area of the Americas.

## Figures and Tables

**Table 1:** Primer and TaqMan probe sequences used in the *Paracoccidioides* multiplex qPCR assay.

Target	Primer and Probe	Primer and Probe Sequences (5′–3′)	Tm (°C)	Final Concentration	Reporter (5′)/Quencher (3′) ^1^
Genus specific	Paracoco-F	CGTATGGGGCTTCGTCACAC	60	900 nM	-/-
	Paracoco-R	CCCTACCTGATCCGAGGTCAAC	60	300 nM	-/-
*P. brasiliensis* complex ^2^	PbraCx-Fam	AAGGTGTCCTCTCTCGATCGACACCCTTC	70	250 nM	FAM/QSY
*P. lutzii*	Plu-Ned	TCGACATCTTCCCCTCTT	70	250 nM	NED/MGB-NFQ
Genus specific	Paracoco-Vic	CCCGCCGGGGTCGATGAGT	70	250 nM	VIC/QSY

^1^ FAM: 6-Carboxyfluorescein, NED: benzofluorotrichlorocarboxy-fluorescein, VIC: 2′-chloro-phenyl-1,4-dichloro-6-carboxyfluorescein, NFQ: non-fluorescent quencher, MGB: minor groove binder. ^2^ Members of the *P. brasiliensis* complex include phylogenetic species S1, PS2, PS3, and PS4.

## Data Availability

The data presented in this study are available within the article and supplementary material.

## Supplementary Materials

The following supporting information can be downloaded at: https://www.mdpi.com/article/10.3390/jof9030358/s1, Supplementary Table S1: *Paracoccidioides* isolates used in this study. Supplementary Table S2: Genomes used in this study for primer design. Supplementary Table S3: DNA samples derived from non-target species, including agents of superficial, subcutaneous, and systemic mycosis in humans and animals. Supplementary Table S4: List of formalin-fixed paraffin-embedded (FFPE) tissue and other biological samples included in the analysis. Supplementary Table S5: Primer-BLAST Results (https://www.ncbi.nlm.nih.gov/tools/primer-blast/ Accessed on 2 January 2023; 13:50 h GMT). Supplementary Figure S1: Alignment of the target region (partial ITS2/28S) for all members of the genus *Paracoccidioides*. The primers (gray boxes) and the probes PbraCx-Fam (yellow box), Plu-Ned (green box), and Paracoco-Vic (blue box) are annotated in the figure. The percentage of conservation and the consensus sequence appears at the alignment’s bottom. In silico analyses show that the primers and probes were designed in highly conserved regions for their respective targets. However, the assay is incompatible with previously published *P. cetii* and *P. loboi* sequences. Supplementary Figure S2: STARD flow diagram of the study population for the (A) formalin-fixed, paraffin-embedded (FFPE) tissue samples and (B) fresh specimens. As a quality control, all DNAs extracted from FFPE samples or fresh clinical specimens were evaluated via PCR using a quadruplex assay targeting nonoverlapping sites in the GAPDH gene (chr12), and samples that yielded 100, 200, 300, and 400 bp amplicons were regarded to be free of PCR inhibitors. Afterward, high-quality DNA samples were subjected to the triplex probe-based qPCR assay and the conventional duplex PCR assay previously described by Pinheiro et al. Supplementary Figure S3: Simple interpretation of qPCR results for singleplex- (orange panel), duplex- (blue panel), and triplex-probe qPCR assays. Regardless of the etiological agent, the Paracoco-Vic probe allows only a generic identification and does not allow the investigation of mixed infections. On the other hand, the duplex assay (PbraCx-Fam and Plu-Ned probes) permits the differentiation of the etiological agents of the *P. brasiliensis* complex and *P. lutzii*, thus allowing the discrimination of mixed infections caused by *P. brasiliensis* complex and *P. lutzii*.
