# Supplementary material for: Development of a Multiplex qPCR Assay for Fast Detection and Differentiation of Paracoccidioidomycosis Agents
Source: J Fungi (Basel). 2023 Mar 15;9(3):358. doi: 10.3390/jof9030358 (PMC10057483; doi:10.3390/jof9030358)
Supplement: Supplementary file 1 [file jof-09-00358-s001.zip › jof-2206119-supplementary.pdf]

Supplementary Files

Table S1. *Paracoccidioides* isolates used in this study.

Isolate code	Genetic Group	Phylogenetic species[1]	qPCR[2]	Conventional PCR[3]	Origin	Ref
EPM01	PS3	*P. restrepiensis*	*P. brasiliensis* complex	*P. brasiliensis* complex	Brazil	[1–3]
EPM09	S1	*P. brasiliensis s. str.*	*P. brasiliensis* complex	*P. brasiliensis* complex	-	[1–3]
EPM10	S1	*P. brasiliensis s. str.*	*P. brasiliensis* complex	*P. brasiliensis* complex	Brazil	[1–3]
EPM16	S1	*P. brasiliensis s. str.*	*P. brasiliensis* complex	*P. brasiliensis* complex	Brazil	[1–3]
EPM18	S1	*P. brasiliensis s. str.*	*P. brasiliensis* complex	*P. brasiliensis* complex	Brazil	[1–3]
EPM30	PS3	*P. restrepiensis*	*P. brasiliensis* complex	*P. brasiliensis* complex	Brazil	[1–3]
EPM33	S1	*P. brasiliensis s. str.*	*P. brasiliensis* complex	*P. brasiliensis* complex	Central America	[1–3]
EPM35	PS2	*P. americana*	*P. brasiliensis* complex	*P. brasiliensis* complex	Uruguay	[1–3]
EPM38	S1	*P. brasiliensis s. str.*	*P. brasiliensis* complex	*P. brasiliensis* complex	Peru	[1–3]
EPM46	S1	*P. brasiliensis s. str.*	*P. brasiliensis* complex	*P. brasiliensis* complex	Argentina	[1–3]
EPM56	S1	*P. brasiliensis s. str.*	*P. brasiliensis* complex	*P. brasiliensis* complex	Venezuela	[1–3]
EPM58	S1	*P. brasiliensis s. str.*	*P. brasiliensis* complex	*P. brasiliensis* complex	Venezuela	[1–3]
EPM61	PS3	*P. restrepiensis*	*P. brasiliensis* complex	*P. brasiliensis* complex	Venezuela	[1–3]
EPM63	S1	*P. brasiliensis s. str.*	*P. brasiliensis* complex	*P. brasiliensis* complex	Venezuela	[1–3]
EPM73	PS4	*P. venezuelensis*	*P. brasiliensis* complex	*P. brasiliensis* complex	Venezuela	[1–3]
EPM74	PS4	*P. venezuelensis*	*P. brasiliensis* complex	*P. brasiliensis* complex	Venezuela	[1–3]
EPM77	PS3	*P. venezuelensis*	*P. brasiliensis* complex	*P. brasiliensis* complex	Colombia	[1–3]
EPM78	PS4	*P. venezuelensis*	*P. brasiliensis* complex	*P. brasiliensis* complex	Venezuela	[1–3]
EPM80	S1	*P. brasiliensis s. str.*	*P. brasiliensis* complex	*P. brasiliensis* complex	Venezuela	[1–3]
EPM81	PS3	*P. restrepiensis*	*P. brasiliensis* complex	*P. brasiliensis* complex	Colombia	[1–3]
EPM84	S1	*P. brasiliensis s. str.*	*P. brasiliensis* complex	*P. brasiliensis* complex	Venezuela	[1–3]
EPM85	S1	*P. brasiliensis s. str.*	*P. brasiliensis* complex	*P. brasiliensis* complex	Peru	[1–3]
EPM87	PS2	*P. americana*	*P. brasiliensis* complex	*P. brasiliensis* complex	Argentina	[1–3]
EPM90	PS2	*P. americana*	*P. brasiliensis* complex	*P. brasiliensis* complex	Argentina	[1–3]
EPM108	S1	*P. brasiliensis s. str.*	*P. brasiliensis* complex	*P. brasiliensis* complex	Peru	[1–3]
EPM112	S1	*P. brasiliensis s. str.*	*P. brasiliensis* complex	*P. brasiliensis* complex	Brazil	[1–3]
EPM113	S1	*P. brasiliensis s. str.*	*P. brasiliensis* complex	*P. brasiliensis* complex	Brazil	[1–3]
EPM118	S1	*P. brasiliensis s. str.*	*P. brasiliensis* complex	*P. brasiliensis* complex	Brazil	[1–3]
EPM122	S1	*P. brasiliensis s. str.*	*P. brasiliensis* complex	*P. brasiliensis* complex	Brazil	[1–3]
EPM127	S1	*P. brasiliensis s. str.*	*P. brasiliensis* complex	*P. brasiliensis* complex	Peru	[1–3]
EPM135	PS2	*P. americana*	*P. brasiliensis* complex	*P. brasiliensis* complex	Brazil	[1–3]
EPM139	S1	*P. brasiliensis s. str.*	*P. brasiliensis* complex	*P. brasiliensis* complex	Brazil	[1–3]
EPM147	*P. lutzii*	*P. lutzii*	*P. lutzii*	*P. lutzii*	Brazil	[1–3]
EPM148	*P. lutzii*	*P. lutzii*	*P. lutzii*	*P. lutzii*	Brazil	[1–3]
EPM152	S1	*P. brasiliensis s. str.*	*P. brasiliensis* complex	*P. brasiliensis* complex	Peru	[1–3]
EPM153	S1	*P. brasiliensis s. str.*	*P. brasiliensis* complex	*P. brasiliensis* complex	Brazil	[1–3]
EPM165	S1	*P. brasiliensis s. str.*	*P. brasiliensis* complex	*P. brasiliensis* complex	Brazil	[1–3]
EPM198	S1	*P. brasiliensis s. str.*	*P. brasiliensis* complex	*P. brasiliensis* complex	-	[1–3]
EPM204	PS2	*P. americana*	*P. brasiliensis* complex	*P. brasiliensis* complex	-	[1–3]
EPM206	*P. lutzii*	*P. lutzii*	*P. lutzii*	*P. lutzii*	Brazil	[1–3]
EPM208	*P. lutzii*	*P. lutzii*	*P. lutzii*	*P. lutzii*	-	[1–3]
EPM213	*P. lutzii*	*P. lutzii*	*P. lutzii*	*P. lutzii*	Brazil	[1–3]
EPM215	PS2	*P. americana*	*P. brasiliensis* complex	*P. brasiliensis* complex	Brazil	[1–3]
EPM223	*P. lutzii*	*P. lutzii*	*P. lutzii*	*P. lutzii*	Brazil	[1–3]
EPM225	*P. lutzii*	*P. lutzii*	*P. lutzii*	*P. lutzii*	Brazil	[1–3]
EPM232	*P. lutzii*	*P. lutzii*	*P. lutzii*	*P. lutzii*	Brazil	[1–3]

**Isolate code**	**Genetic Group**	**Phylogenetic species**[1]	**qPCR**[2]	**Conventional PCR**[3]	**Origin**	**Ref**
EPM239	*P. lutzii*	*P. lutzii*	*P. lutzii*	*P. lutzii*	Brazil	[1–3]
EPM242	*P. lutzii*	*P. lutzii*	*P. lutzii*	*P. lutzii*	Brazil	[1–3]
EPM249	S1	*P. brasiliensis s. str.*	*P. brasiliensis* complex	*P. brasiliensis* complex	Brazil	[1–3]
EPM250	S1	*P. brasiliensis s. str.*	*P. brasiliensis* complex	*P. brasiliensis* complex	Brazil	[1–3]
EPM254	S1	*P. brasiliensis s. str.*	*P. brasiliensis* complex	*P. brasiliensis* complex	Brazil	[1–3]
EPM255	S1	*P. brasiliensis s. str.*	*P. brasiliensis* complex	*P. brasiliensis* complex	Brazil	[1–3]
EPM256	S1	*P. brasiliensis s. str.*	*P. brasiliensis* complex	*P. brasiliensis* complex	Brazil	[1–3]
EPM258	S1	*P. brasiliensis s. str.*	*P. brasiliensis* complex	*P. brasiliensis* complex	Brazil	[1–3]
EPM259	S1	*P. brasiliensis s. str.*	*P. brasiliensis* complex	*P. brasiliensis* complex	Brazil	[1–3]
EPM260	S1	*P. brasiliensis s. str.*	*P. brasiliensis* complex	*P. brasiliensis* complex	Brazil	[1–3]
EPM261	S1	*P. brasiliensis s. str.*	*P. brasiliensis* complex	*P. brasiliensis* complex	Brazil	[1–3]
EPM263	S1	*P. brasiliensis s. str.*	*P. brasiliensis* complex	*P. brasiliensis* complex	Brazil	[1–3]
EPM264	S1	*P. brasiliensis s. str.*	*P. brasiliensis* complex	*P. brasiliensis* complex	Brazil	[1–3]
EPM271	S1	*P. brasiliensis s. str.*	*P. brasiliensis* complex	*P. brasiliensis* complex	Brazil	[1–3]
EPM273	S1	*P. brasiliensis s. str.*	*P. brasiliensis* complex	*P. brasiliensis* complex	Brazil	[1–3]
EPM276	S1	*P. brasiliensis s. str.*	*P. brasiliensis* complex	*P. brasiliensis* complex	Brazil	[1–3]
EPM283	*P. lutzii*	*P. lutzii*	*P. lutzii*	*P. lutzii*	Brazil	[1–3]
EPM284	*P. lutzii*	*P. lutzii*	*P. lutzii*	*P. lutzii*	Brazil	[1–3]
EPM285	*P. lutzii*	*P. lutzii*	*P. lutzii*	*P. lutzii*	Brazil	[1–3]
EPM287	*P. lutzii*	*P. lutzii*	*P. lutzii*	*P. lutzii*	Brazil	[1–3]
EPM288	*P. lutzii*	*P. lutzii*	*P. lutzii*	*P. lutzii*	Brazil	[1–3]
EPM289	*P. lutzii*	*P. lutzii*	*P. lutzii*	*P. lutzii*	Brazil	[1–3]
EPM290	S1	*P. brasiliensis s. str.*	*P. brasiliensis* complex	*P. brasiliensis* complex	Brazil	[1–3]
EPM291	S1	*P. brasiliensis s. str.*	*P. brasiliensis* complex	*P. brasiliensis* complex	-	[1–3]
EPM292	S1	*P. brasiliensis s. str.*	*P. brasiliensis* complex	*P. brasiliensis* complex	Brazil	[1–3]
EPM293	*P. lutzii*	*P. lutzii*	*P. lutzii*	*P. lutzii*	Brazil	[1–3]
EPM294	*P. lutzii*	*P. lutzii*	*P. lutzii*	*P. lutzii*	Brazil	[1–3]
EPM295	*P. lutzii*	*P. lutzii*	*P. lutzii*	*P. lutzii*	Brazil	[1–3]
EPM296	*P. lutzii*	*P. lutzii*	*P. lutzii*	*P. lutzii*	Brazil	[1–3]
EPM297	*P. lutzii*	*P. lutzii*	*P. lutzii*	*P. lutzii*	Brazil	[1–3]
EPM298	S1	*P. brasiliensis s. str.*	*P. brasiliensis* complex	*P. brasiliensis* complex	Brazil	[1–3]

Table S2. Genomes used in this study for primer design.

Genome	Genetic Group	Phylogenetic species	Source	Origin	BioSample	Ref
Pb18	S1	*P. brasiliensis s. str.*	Chronic PCM	Brazil	SAMN05171520	[4]
PbCaz	S1	*P. brasiliensis s. str.*	Acute PCM	Argentina	SAMN05171521	[4]
Pb113	S1	*P. brasiliensis s. str.*	PCM	Amazonas	SAMN05171522	[4]
PbBlo	S1	*P. brasiliensis s. str.*	PCM	Brazil	SAMN05171523	[4]
MS1	S1	*P. brasiliensis s. str.*	PCM	Brazil	SAMN05171524	[4]
D03	S1	*P. brasiliensis s. str.*	PCM	Brazil	SAMN05171525	[4]
MS2	S1	*P. brasiliensis s. str.*	PCM	Brazil	SAMN05171526	[4]
Pb1445	S1	*P. brasiliensis s. str.*	Chronic PCM	Argentina	SAMN05171527	[4]
Pb337	S1	*P. brasiliensis s. str.*	*D. novemcinctus*	Brazil	SAMN05171528	[4]
Pb66	S1	*P. brasiliensis s. str.*	PCM	Brazil	SAMN05171529	[4]
PbBer	S1	*P. brasiliensis s. str.*	PCM	Argentina	SAMN05171530	[4]
D02	S1	*P. brasiliensis s. str.*	PCM	Brazil	SAMN05171531	[4]
T1F1	S1	*P. brasiliensis s. str.*	*D. novemcinctus*	Brazil	SAMN05171532	[4]
T15N1	S1	*P. brasiliensis s. str.*	*D. novemcinctus*	Brazil	SAMN05171533	[4]
T16B1	S1	*P. brasiliensis s. str.*	*D. novemcinctus*	Brazil	SAMN05171534	[4]
Pb300	PS4	*P. venezuelensis*	Soil	Venezuela	SAMN03787412	[4]
EPM83	PS3	*P. restrepiensis*	Chronic PCM	Colombia	SAMN05171535	[4]
PB339	PS3	*P. restrepiensis*	PCM	Brazil	SAMN05171536	[4]
Pb60855	PS3	*P. restrepiensis*	Chronic PCM	Colombia	SAMN05171537	[4]
PbBac	PS3	*P. restrepiensis*	PCM	Colombia	SAMN05171538	[4]
PbCab	PS3	*P. restrepiensis*	C. centralis	Colombia	SAMN05171539	[4]
PbCnh	PS3	*P. restrepiensis*	Chronic PCM	Colombia	SAMN03793109	[4]
PbJam	PS3	*P. restrepiensis*	Chronic PCM	Colombia	SAMN05171540	[4]
Pb02	PS2	*P. americana*	Chronic PCM	Venezuela	SAMN05171541	[4]
Pb03	PS2	*P. americana*	Chronic PCM	Brazil	SAMN05171542	[4]
Pb262	PS2	*P. americana*	Dog food	Brazil	SAMN05171543	[4]
T10B1	PS2	*P. americana*	*D. novemcinctus*	Brazil	SAMN05171544	[4]
Pb01	*P. lutzii*	*P. lutzii*	PCM	Brazil	SAMN05171545	[4]
Pl1578	*P. lutzii*	*P. lutzii*	PCM	Brazil	SAMN05171546	[4]
ED01	*P. lutzii*	*P. lutzii*	PCM	Brazil	SAMN05171547	[4]
PlEE	*P. lutzii*	*P. lutzii*	PCM	Brazil	SAMN05171548	[4]

Table S3. DNA samples derived from non-target species, including agents of superficial, subcutaneous, and systemic mycosis in humans and animals.

Isolate	Species	Origin	Reference
Ss06	*Sporothrix globosa*	Brazil	[5]
Ss54	*Sporothrix brasiliensis*	Brazil	[5]
Ss126	*Sporothrix schenckii*	Brazil	[5]
Ss132	*Sporothrix mexicana*	Brazil	[5]
Hc1	*Histoplasma capsulatum*	Brazil	[5]
Hc2	*Histoplasma capsulatum*	Brazil	[5]
Hc3	*Histoplasma capsulatum*	Brazil	[5]
Hc4	*Histoplasma capsulatum*	Brazil	[5]
AZ	*Aspergillus* spp.	Brazil	[5]
11p	*Chrysosporium* spp.	Brazil	[5]
Deb	*Debaryomyces* spp.	Brazil	[5]
Ck	*Candida krusei*	Brazil	[5]
Ca	*Candida albicans*	Brazil	[5]
Cp	*Candida parapsilosis*	Brazil	[5]
Co ATCC	*Candida orthopsilosis*	Brazil	[5]
Co CECT	*Candida orthopsilosis*	Brazil	This study
Cm	*Candida metapsilosis*	Brazil	[5]
CN55	*Cryptococcus neoformans*	Brazil	This study
C659	*Cryptococcus gattii*	Brazil	This study
C660	*Cryptococcus gattii*	Brazil	[5]
C63	*Coccidioides immitis*	Brazil	[5]
C64	*Coccidioides posadasii*	Brazil	[5]
C65	*Coccidioides immitis*	Brazil	[5]
C66	*Coccidioides posadasii*	Brazil	[5]
F35	*Fusarium oxysporum*	Brazil	[5]
F73	*Fusarium solani*	Brazil	[5]
F131	*Fusarium fujikuroi*	Brazil	This study
F140	*Fusarium dimerum*	Brazil	[5]

Table S4. List of formalin-fixed paraffin-embedded (FFPE) tissue and other biological samples included in the analysis.

Group	Code	Year	Organ/Specimen	Gender	Age	Origin
FFPE-PCM	FFPE 01	2018	Lung	Male	N/A	São Paulo, SP, Brazil
FFPE-PCM	FFPE 05	2010	Lymph node	Male	42	Juiz de Fora, MG, Brazil
FFPE-PCM	FFPE 06	2011	Lymph node	Male	13	São Paulo, SP, Brazil
FFPE-PCM	FFPE 07	2011	Lymph node	Male	13	São Paulo, SP, Brazil
FFPE-PCM	FFPE 08	2019	Oral mucosa	Male	50	São Carlos, SP, Brazil
FFPE-PCM	FFPE 09	2019	Oral mucosa	Male	50	São Carlos, SP, Brazil
FFPE-PCM	FFPE 10	2019	Skin	Male	58	São Carlos, SP, Brazil
FFPE-PCM	FFPE 11	2014	Duodenum	Female	35	São Carlos, SP, Brazil
FFPE-PCM	FFPE 12	2019	Kidney	Male	N/A	São Paulo, SP, Brazil
FFPE-PCM	FFPE 13	2011	Tibia	Male	19	São Paulo, SP, Brazil
FFPE-PCM	FFPE 14	2011	Tibia	Male	18	São Paulo, SP, Brazil
FFPE-PCM	FFPE 15	2018	Lymph node	Male	55	São Paulo, SP, Brazil
FFPE-PCM	FFPE 16	2016	Lung	Male	64	São Paulo, SP, Brazil
FFPE-PCM	FFPE 17	2011	Lymph node	Male	14	São Paulo, SP, Brazil
FFPE-PCM	FFPE 20	2010	Skin	Male	66	São Paulo, SP, Brazil

FFPE-PCM	FFPE 21	2020	Skin	Male	30	São Carlos, SP, Brazil
Fresh specimen-PCM	Sputum 01	2021	Sputum	Male	41	São Paulo, SP, Brazil
Fresh specimen-PCM	Biopsy 01	2021	Skin biopsy	Male	41	São Paulo, SP, Brazil
Fresh specimen-PCM	Biopsy 02	2022	Skin biopsy	Male	N/A	Brazil
Fresh specimen-PCM	Biopsy 03	2015	Lymph node	Male	70	Felisburgo, MG, Brazil
Fresh specimen-PCM	CSF 01	2022	CSF	Male	N/A	Brazil
Fresh specimen-PCM	BAL 01	2021	BAL	Female	51	São Paulo, SP, Brazil
Fresh specimen-PCM	BAL 02	2021	BAL	Female	22	São Paulo, SP, Brazil
Fresh specimen-PCM	BAL 03	2022	BAL	Male	68	São Paulo, SP, Brazil
Fresh specimen-PCM	BAL 04	2022	BAL	Female	73	São Paulo, SP, Brazil

CSF: Cerebrospinal Fluid; BAL: Bronchoalveolar lavage. N/A: Not available.

Table S5. Primer-BLAST Results (https://www.ncbi.nlm.nih.gov/tools/primer-blast/; Accessed on 2 January 2023; 13:50 h GMT).

Search Parameters and Other Details	
Number of Blast hits analyzed	52755
Entrez query	
Min total mismatches	2
Min 3' end mismatches	2
Defined 3' end region length	5
Mismatch threshold to ignore targets	1
Max target size	4000
Max number of Blast target sequences	50000
Blast E value	30000
Blast word size	7
Max candidate primer pairs	500
Min PCR product size	62
Max PCR product size	1000
Min Primer size	15
Opt Primer size	20
Max Primer size	25
Min Tm	57
Opt Tm	60
Max Tm	63
Max Tm difference	3
Repeat filter	AUTO
Low complexity filter	Yes

Primer pair 1

Primers	Sequence (5'->3')	Length	Tm	GC%	Self complementarity	Self 3' complementarity
**Forward**	CGTATGGGGCTTCGTCACAC	20	61.08	60.00	3.00	1.00
**Reverse**	CCCTACCTGATCCGAGGTCAAC	22	61.86	59.09	4.00	2.00

Products on target templates

>XR_007137646.1 Paracoccidioides brasiliensis Pb18 28S ribosomal RNA (PADG_12520), partial rRNA

```
product length = 143
Forward primer  1     CGTATGGGGCTTCGTCACAC  20
Template        247   ....................  266
Reverse primer  1     CCCTACCTGATCCGAGGTCAAC  22
Template        389   ......................  368
```

>XR_007137644.1 Paracoccidioides brasiliensis Pb18 28S ribosomal RNA (PADG_12518), rRNA

```
product length = 143
Forward primer  1     CGTATGGGGCTTCGTCACAC  20
Template        247   ....................  266
Reverse primer  1     CCCTACCTGATCCGAGGTCAAC  22
Template        389   ......................  368
```

>XR_007137643.1 Paracoccidioides brasiliensis Pb18 28S ribosomal RNA (PADG_12517), rRNA

```
product length = 143
Forward primer  1     CGTATGGGGCTTCGTCACAC  20
Template        247   ....................  266
Reverse primer  1     CCCTACCTGATCCGAGGTCAAC  22
Template        389   ......................  368
```

>XR_007137641.1 Paracoccidioides brasiliensis Pb18 28S ribosomal RNA (PADG_12514), rRNA

```
product length = 143
Forward primer  1     CGTATGGGGCTTCGTCACAC  20
Template        247   ....................  266
Reverse primer  1     CCCTACCTGATCCGAGGTCAAC  22
Template        389   ......................  368
```

>XR_007137633.1 Paracoccidioides brasiliensis Pb18 28S ribosomal RNA (PADG_12094), rRNA

```
product length = 143
Forward primer  1     CGTATGGGGCTTCGTCACAC  20
Template        247   ....................  266
Reverse primer  1     CCCTACCTGATCCGAGGTCAAC  22
Template        389   ......................  368
```

>XR_007137631.1 Paracoccidioides brasiliensis Pb18 28S ribosomal RNA (PADG_12092), rRNA

```
product length = 143
Forward primer  1     CGTATGGGGCTTCGTCACAC  20
Template        247   ....................  266
Reverse primer  1     CCCTACCTGATCCGAGGTCAAC  22
Template        389   ......................  368
```

>XR_007137629.1 Paracoccidioides brasiliensis Pb18 28S ribosomal RNA (PADG_12089), rRNA

```
product length = 142
Forward primer  1    CGTATGGGGCTTCGTCACAC  20
Template        247  ....................  266
Reverse primer  1    CCCTACCTGATCCGAGGTCAAC  22
Template        388  ......................  367
```

>MH860706.1 Paracoccidioides brasiliensis culture CBS:372.73 strain CBS 372.73 small subunit ribosomal RNA gene, partial sequence; internal transcribed spacer 1, 5.8S ribosomal RNA gene, and internal transcribed spacer 2, complete sequence; and large subunit ribosomal RNA gene, partial sequence

```
product length = 143
Forward primer  1    CGTATGGGGCTTCGTCACAC  20
Template        488  ....................  507
Reverse primer  1    CCCTACCTGATCCGAGGTCAAC  22
Template        630  ......................  609
```

>MN271899.2 Paracoccidioides brasiliensis isolate 104 5.8S ribosomal RNA gene, partial sequence; internal transcribed spacer 2, complete sequence; and large subunit ribosomal RNA gene, partial sequence

```
product length = 143
Forward primer  1    CGTATGGGGCTTCGTCACAC  20
Template        173  ....................  192
Reverse primer  1    CCCTACCTGATCCGAGGTCAAC  22
Template        315  ......................  294
```

>MN271901.1 Paracoccidioides brasiliensis isolate 22 5.8S ribosomal RNA gene, partial sequence; internal transcribed spacer 2, complete sequence; and large subunit ribosomal RNA gene, partial sequence

```
product length = 143
Forward primer  1    CGTATGGGGCTTCGTCACAC  20
Template        235  ....................  254
Reverse primer  1    CCCTACCTGATCCGAGGTCAAC  22
Template        377  ......................  356
```

>MN271900.1 Paracoccidioides brasiliensis isolate 19 5.8S ribosomal RNA gene, partial sequence; internal transcribed spacer 2, complete sequence; and large subunit ribosomal RNA gene, partial sequence

```
product length = 143
Forward primer  1    CGTATGGGGCTTCGTCACAC  20
Template        235  ....................  254
Reverse primer  1    CCCTACCTGATCCGAGGTCAAC  22
Template        377  ......................  356
```

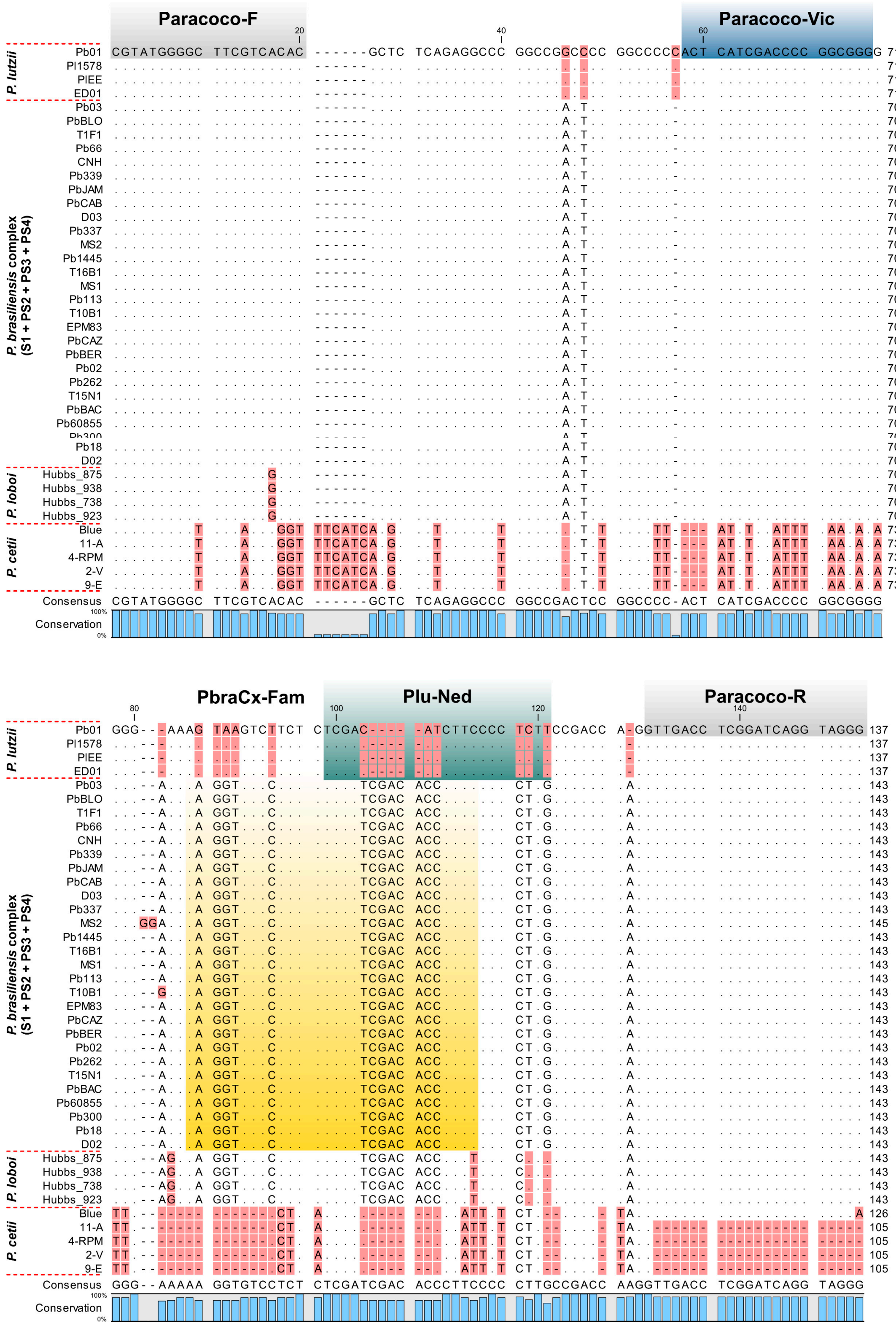

Figure S1. Alignment of the target region (partial ITS2/28S) for all members of the genus *Paracoccidioides* [4,6,7]. The primers (gray boxes) and the probes PbraCx-Fam (yellow box), Plu-Ned (green box), and Paracoco-Vic (blue box) are annotated in the figure. The percentage of conservation and the consensus sequence appears at the alignment's bottom. *In silico* analyses show that the primers and probes were designed in highly conserved regions for their respective targets. However, the assay is incompatible with previously published *P. cetii* and *P. loboi* sequences [6,7].

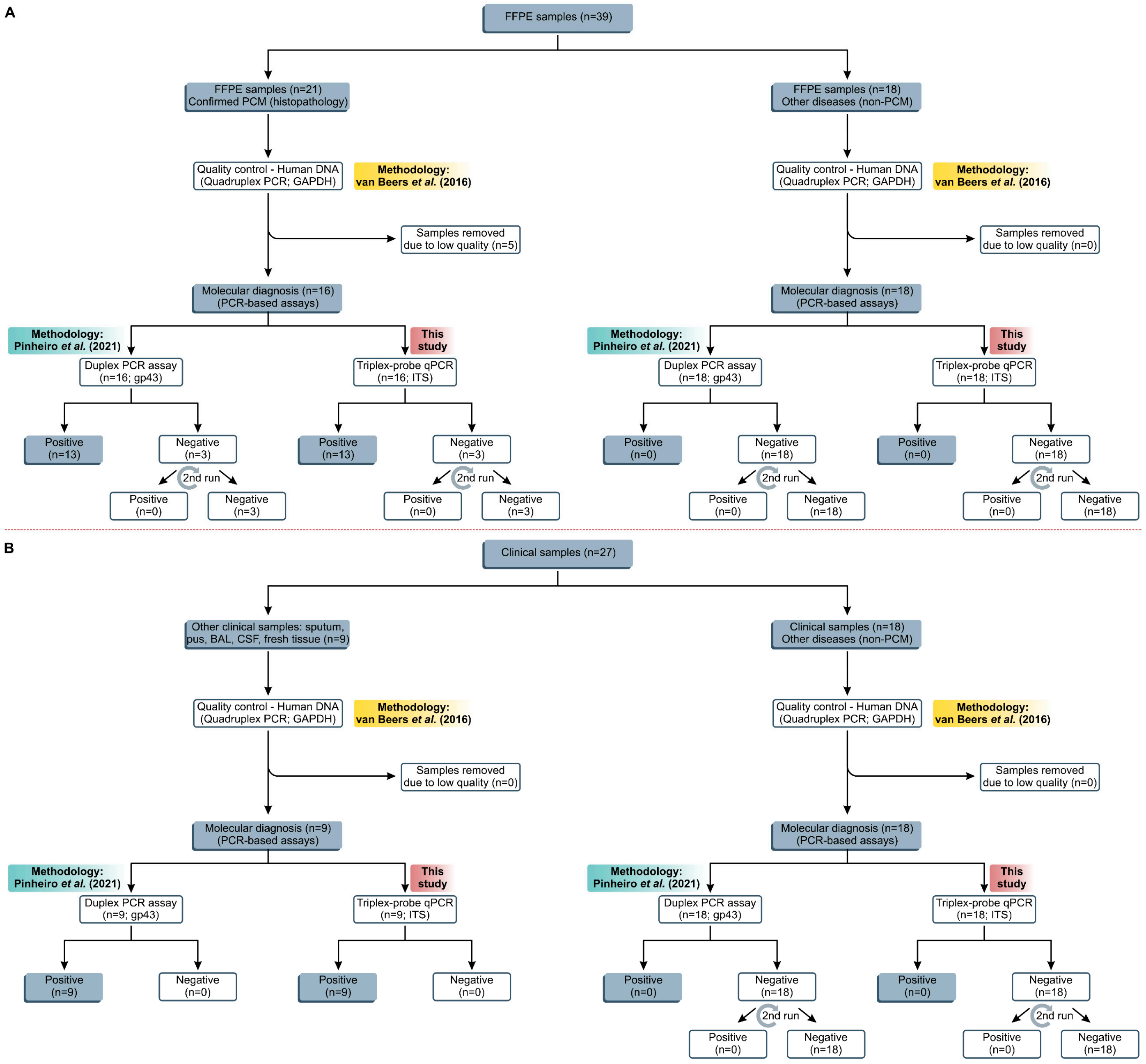

Figure S2. STARD flow diagram of the study population for the (A) formalin-fixed, paraffin-embedded (FFPE) tissue samples and (B) fresh specimens. As a quality control, all DNAs extracted from FFPE samples or fresh clinical specimens were evaluated via PCR using a quadruplex assay targeting nonoverlapping sites in the GAPDH gene (chr12) [8], and samples that yielded 100, 200, 300, and 400 bp amplicons were regarded to be free of PCR inhibitors. Afterward, high-quality DNA samples were subjected to the triplex probe-based qPCR assay and the conventional duplex PCR assay previously described by Pinheiro *et al.* [3].

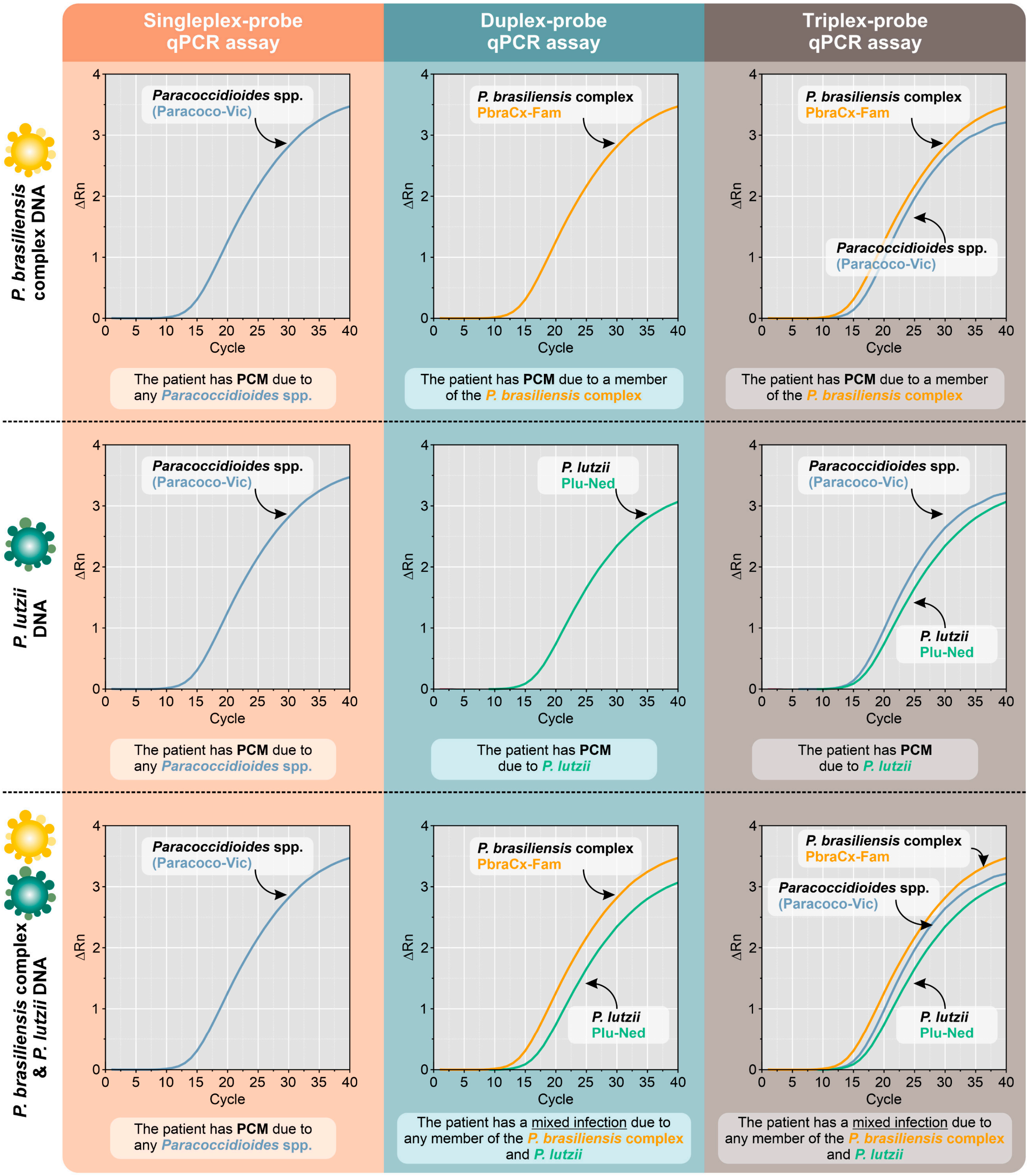

Figure S3. Simple interpretation of qPCR results for singleplex (orange panel), duplex (blue panel), and triplex-probe qPCR assays (grey panel). Regardless of the etiological agent, the Paracoco-Vic probe allows only a generic identification and does not allow the investigation of mixed infections. On the other hand, the duplex assay (PbraCx-Fam and Plu-Ned probes) permits the differentiation of the etiological agents of the *P. brasiliensis* complex and *P. lutzii*, thus allowing the discrimination of mixed infections caused by *P. brasiliensis* complex and *P. lutzii*.

Supplementary References

1. Roberto, T.N.; Rodrigues, A.M.; Hahn, R.C.; de Camargo, Z.P. Identifying *Paracoccidioides* phylogenetic species by PCR-RFLP of the alpha-tubulin gene. *Med. Mycol.* **2016**, *54*, 240-247, doi:10.1093/mmy/myv083.
2. Roberto, T.N.; De Carvalho, J.A.; Beale, M.A.; Hagen, F.; Fisher, M.C.; Hahn, R.C.; de Camargo, Z.P.; Rodrigues, A.M. Exploring genetic diversity, population structure, and phylogeography in *Paracoccidioides* species using AFLP markers. *Stud. Mycol.* **2021**, *100*, 100131, doi:10.1016/j.simyco.2021.100131.
3. Pinheiro, B.G.; Pôssa, A.P.; Della Terra, P.P.D.; de Carvalho, J.A.d.; Ricci, G.; Nishikaku, A.S.; Hahn, R.C.; Camargo, Z.P.d.; Rodrigues, A.M. A new duplex PCR-assay for the detection and identification of *Paracoccidioides* species. *J. Fungi* **2021**, *7*, 169, doi:10.3390/jof7030169.
4. Muñoz, J.F.; Farrer, R.A.; Desjardins, C.A.; Gallo, J.E.; Sykes, S.; Sakthikumar, S.; Misas, E.; Whiston, E.A.; Bagagli, E.; Soares, C.M.; et al. Genome diversity, recombination, and virulence across the major lineages of *Paracoccidioides*. *mSphere* **2016**, *1*, e00213-00216, doi:10.1128/mSphere.00213-16.
5. Della Terra, P.P.; Gonsales, F.F.; de Carvalho, J.A.; Hagen, F.; Kano, R.; Bonifaz, A.; Camargo, Z.P.; Rodrigues, A.M. Development and evaluation of a multiplex qPCR assay for rapid diagnostics of emerging sporotrichosis. *Transbound. Emerg. Dis.* **2022**, *69*, e704-e716, doi:10.1111/tbed.14350.
6. Vilela, R.; Huebner, M.; Vilela, C.; Vilela, G.; Pettersen, B.; Oliveira, C.; Mendoza, L. The taxonomy of two uncultivated fungal mammalian pathogens is revealed through phylogeny and population genetic analyses. *Sci. Rep.* **2021**, *11*, 18119, doi:10.1038/s41598-021-97429-7.
7. Vilela, R.; Rosa, P.S.; Belone, A.F.; Taylor, J.W.; Diorio, S.M.; Mendoza, L. Molecular phylogeny of animal pathogen *Lacazia loboi* inferred from rDNA and DNA coding sequences. *Mycol. Res.* **2009**, *113*, 851-857, doi:10.1016/j.mycres.2009.04.007.
8. van Beers, E.H.; Joosse, S.A.; Ligtenberg, M.J.; Fles, R.; Hogervorst, F.B.L.; Verhoef, S.; Nederlof, P.M. A multiplex PCR predictor for aCGH success of FFPE samples. *Br. J. Cancer* **2006**, *94*, 333–337. https://doi.org/10.1038/sj.bjc.6602889.
